# Use of high flow nasal cannula in patients with acute respiratory failure in general wards under intensivists supervision: a single center observational study

**DOI:** 10.1186/s12931-022-02090-x

**Published:** 2022-06-26

**Authors:** Sebastiano Maria Colombo, Vittorio Scaravilli, Andrea Cortegiani, Nadia Corcione, Amedeo Guzzardella, Luca Baldini, Elisa Cassinotti, Ciro Canetta, Stefano Carugo, Cinzia Hu, Anna Ludovica Fracanzani, Ludovico Furlan, Maria Chiara Paleari, Alessandro Galazzi, Paola Tagliabue, Flora Peyvandi, Francesco Blasi, Giacomo Grasselli

**Affiliations:** 1 Department of Anaesthesia and Intensive Care Medicine, Fondazione IRCCS Ca’ Granda Ospedale Maggiore Policlinico, Via Sforza, 35, 20122 Milan, Lombardia Italy; 2 Department of Pathophysiology and Transplantation, University of Milan, Milan, Lombardia Italy; 3 Department of Biomedical, Surgical and Dental Sciences, University of Milan, Milan, Lombardia Italy; 4 Department of Surgical, Oncological and Oral Science (Di.Chir.On.S.), University of Palermo, Palermo, Sicilia Italy; 5 Department of Anesthesia, Intensive Care and Emergency, Policlinico Paolo Giaccone, Palermo, Sicilia Italy; 6 Respiratory Unit and Cystic Fibrosis Adult Center, Fondazione IRCCS Ca’ Granda Ospedale Maggiore Policlinico, Milan, Lombardia Italy; 7 Azienda Ospedaliera Antonio Caldarelli, Interventional Pulmunology, Naples, Campania Italy; 8 Department of Internal Medicine, Fondazione IRCCS Ca’ Granda Ospedale Maggiore Policlinico, Milan, Lombardia Italy; 9 Department of Oncology and Hemato-oncology, University of Milan, Milan, Lombardia Italy; 10 Department of Surgery, Fondazione IRCCS Ca’ Granda Ospedale Maggiore Policlinico, Milan, Lombardia Italy; 11 Department of Clinical Sciences and Community Health, University of Milan, Milan, Lombardia Italy; 12 Unit of Internal Medicine and Metabolic Disease, Fondazione IRCCS Ca’ Granda Ospedale Maggiore Policlinico, Milan, Lombardia Italy; 13 Healthcare Profession Department, Fondazione IRCCS Ca’ Granda Ospedale Maggiore Policlinico, Milan, Lombardia Italy; 14 Internal Medicine Department and Respiratory Medicine Unit, Fondazione IRCCS Ca’ Granda Ospedale Maggiore Policlinico, Milan, Lombardia Italy

**Keywords:** HFNC, ARF, AHRF, ICU-supervision, General-wards, Safety

## Abstract

**Background:**

Few data exist on high flow nasal cannula (HFNC) use in patients with acute respiratory failure (ARF) admitted to general wards.

**Rationale and objectives:**

To retrospectively evaluate feasibility and safety of HFNC in general wards under the intensivist-supervision and after specific training.

**Methods:**

Patients with ARF (dyspnea, respiratory rate-RR > 25/min, 150 < PaO_2_/FiO_2_ < 300 mmHg during oxygen therapy) admitted to nine wards of an academic hospital were included. Gas-exchange, RR, and comfort were assessed before HFNC and after 2 and 24 h of application.

**Results:**

150 patients (81 male, age 74 [60–80] years, SOFA 4 [2–4]), 123 with de-novo ARF underwent HFNC with flow 60 L/min [50–60], FiO_2_ 50% [36–50] and temperature 34 °C [31–37]. HFNC was applied a total of 1399 days, with a median duration of 7 [3–11] days. No major adverse events or deaths were reported. HFNC did not affect gas exchange but reduced RR (25–22/min at 2–24 h, p < 0.001), and improved Dyspnea Borg Scale (3–1, p < 0.001) and comfort (3–4, p < 0.001) after 24 h. HFNC failed in 20 patients (19.2%): 3 (2.9%) for intolerance, 14 (13.4%) escalated to NIV/CPAP in the ward, 3 (2.9%) transferred to ICU. Among these, one continued HFNC, while the other 2 were intubated and they both died. Predictors of HFNC failure were higher Charlson’s Comorbidity Index (OR 1.29 [1.07–1.55]; p = 0.004), higher APACHE II Score (OR 1.59 [1.09–4.17]; p = 0.003), and cardiac failure as cause of ARF (OR 5.26 [1.36–20.46]; p = 0.02).

**Conclusion:**

In patients with mild-moderate ARF admitted to general wards, the use of HFNC after an initial training and daily supervision by intensivists was feasible and seemed safe. HFNC was effective in improving comfort, dyspnea, and respiratory rate without effects on gas exchanges.

*Trial registration* This is a single-centre, noninterventional, retrospective analysis of clinical data.

**Supplementary Information:**

The online version contains supplementary material available at 10.1186/s12931-022-02090-x.

## Highlights

- Few data exist about feasibility and safety of HFNC outside the ICU.
- This study retrospectively evaluates safety, feasibility and efficacy of HFNC used in general wards under intensivist-supervision and specific training courses.
- 150 patients with mild-moderate ARF admitted to general wards were included.
- HFNC demonstrated to be feasible and potentially safe (no deaths or major adverse events in the wards were reported).
- HFNC was effective in improving comfort, dyspnea and RR without effects on gas exchange.
- Failure was recorded in 20 patients (19.2%): 14 escalations to CPAP/NIV and 3 transfers to ICU.

**Supplementary Information:**

The online version contains supplementary material available at 10.1186/s12931-022-02090-x.

## Background

High flow nasal cannula (HFNC) has become an established form of non-invasive respiratory support for acute hypoxemic respiratory failure (AHRF) patients. HFNC is a relatively recent respiratory support technique which delivers a heated and humidified high flow mix (up to 60 L/min) at controlled concentration of oxygen via the nasal route. Although the widespread clinical use and solid evidence [1] on the application of HFNC in adult patients in Intensive Care Units (ICU) and Emergency Departments, limited data exist in patients admitted to general wards. During the Coronavirus-19 disease (COVID-19) pandemic, the use of HFNC as respiratory support in patients outside the ICU increased enormously. However, outside the context of the COVID-19 pandemic [2], limited data are available on the use of HFNC in patients with AHRF admitted to general wards [3–6]. This study aimed to evaluate the feasibility, safety, and efficacy of HFNC in general wards after specific training by and under the supervision of the intensivists.

## Methods

### Centre and ethics

This is a single-centre, noninterventional, retrospective analysis of clinical data. The local Ethics Committee approved the study, and informed consent was waived due to the retrospective nature of the analyses.

In September 2017 our hospital developed an internal protocol to guide the application of HFNC in the wards and the subsequent monitoring by an intensivist. The protocol was adopted in nine hospital wards (three General Medicine wards, Pulmonology, Cardiology, Emergency Medicine, Emergency Surgery, Haematology and Neurology) of the Fondazione IRCCS Ca’ Granda Ospedale Maggiore Policlinico of Milan, Italy. Participating wards were equipped with AIRVO 2® devices (Fisher & Paykel Healthcare, Auckland, New Zealand). In addition, specific training courses for the medical and nursing staff were conducted by an ICU physician and an ICU nurse in each ward to review indications, contraindications, and technical aspects of HFNC therapy. Details about the training are available in the Additional files 1, 2, 3. The protocol was applied regularly from November 2017 to December 2019.

### Patients

Patients were considered potentially eligible for treatment with HFNC if they had mild-to-moderate purely hypoxemic (AHRF) or mixed hypoxemic-hypercapnic respiratory failure (AMRF), defined as dyspnea or tachypnea (respiratory rate > 25 breaths/min) associated either with:

1. An arterial partial pressure of oxygen to inspiratory oxygen fraction ratio (PaO_2_/FiO_2_) between 150 and 300 mmHg during standard oxygen therapy (i.e., low-flow nasal cannula, Venturi mask)—AHRF; or
2. Hypoxemia defined as above *and* an arterial partial pressure of carbon dioxide (PaCO_2_) > 45 mmHg with arterial pH > 7.30 during standard oxygen therapy—AMRF [7].

### Study protocol

**Fig. 1:**
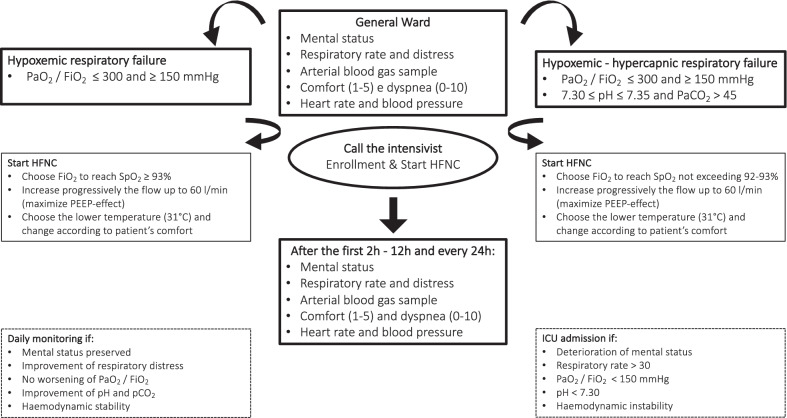
Study flowchart for application of HFNC in general wards under ICU-physician surveillance. *HFNC* high flow nasal cannula; *FiO*_*2*_ inspired fraction of O_2_; *PaO*_*2*_ arterial partial pressure of O_2_; *PaCO*_*2*_ arterial partial pressure of CO_2_; *SpO*_*2*_ peripheral saturation of Hb; *PEEP* positive end-expiratory pressure; *ICU* Intensive Care Unit

Whenever the general ward physicians identified an eligible patient, the ICU Outreach Team was consulted to confirm the indication, and HFNC was started. Airflow was increased progressively up to 60 L/min, with a fraction of inspired oxygen (FiO_2_) titrated to maintain pulse oximetry (SpO_2_) of at least 94% in patients with AHRF or not exceeding 93% in patients with AMRF. The temperature was set according to the patient’s comfort. The internal protocol is presented in Fig. 1.

Exclusion criteria for HFNC application were: age < 18 years old; Glasgow Coma Scale (GCS) ≤ 12; contraindication to HFNC use (i.e., nose-surgery within 3 months); hemodynamic instability (i.e., hypotension with mean arterial pressure < 60 mmHg despite adequate volume resuscitation, infusion of vasoactive drugs or new onset of cardiac arrhythmias); need for immediate intubation (i.e., impending respiratory arrest, inability to protect the airway, shock).

Based on age, comorbidities, etiology of ARF, and actual clinical conditions, patients were assigned by the intensivist to one of the following three categories: (1) patients candidate to *Full-code resuscitation*; (2) *Do-Not-Intubate* (DNI): patients not eligible for ICU admission and endotracheal intubation but candidate to an eventual escalation of the ventilatory support to Continuous Positive Airways Pressure (CPAP) or Non-Invasive-Ventilation (NIV) in the ward; (3) *End-Of-Life* (EOL): patients with terminal diseases in whom HFNC was applied for a palliative purpose and thus not candidate to any escalation of ventilatory support.

The following baseline clinical characteristics were collected before HFNC therapy: demographics, comorbidities, reason for hospitalization, cause of ARF, Sequential Organ Failure Assessment (SOFA) score, Acute Physiologic Assessment and Chronic Health Evaluation (APACHE II score) and Charlson’s Comorbidity Index. An arterial blood gas analysis was performed immediately before HFNC application, then after 2 and 24 h of treatment and subsequently when clinically indicated. After the first 2 h of HFNC and then once daily (or more frequently if clinically indicated), an intensivist of the Outreach Team visited each patient and recorded on a dedicated form the following parameters: respiratory rate, pulse oximetry, comfort (assessed through a visual analogic scale) and Borg Scale dyspnea score. Attending physicians were instructed to immediately consult the Outreach Team in case of any of the following: worsening of mental status; respiratory rate ≥ 30 breaths/min; PaO_2_/FiO_2_ < 150 mmHg; pH < 7.30; hemodynamic instability.

As per hospital procedures, each patient has been daily evaluated by the ward attending physician, while vital signs (i.e., level of consciousness, respiratory rate, oxygen saturation, heart rate, blood pressure, diuresis and temperature) were recorded at least once per shift (three times per day) by the nursing staff.

### Outcomes

Aim of the study was to evaluate feasibility, safety and efficacy of the application of HFNC in patients admitted to general hospital wards under the supervision of an intensivist. Safety and feasibility were defined taking into account the patient tolerance to HFNC, the occurrence of adverse events or clinical complications related to the application of the procedure, or to malfunctions/technical issues. In particular *adverse events* were defined as follows: intolerance to the device, malfunction of the device, technical issues in starting the device and or during its use (i.e., sudden stop in delivering high-flow oxygen therapy, lack in humidification of the gas flow, any use in contrast to the product user manual and device declaration of conformity and use), need of respiratory support escalation due to HFNC malfunction/no-function.

Efficacy was assessed based on the effect of HFNC on gas exchange, comfort, dyspnea, respiratory rate, ROX Index (ratio between SpO_2_/FiO_2_ and respiratory rate) and on the need of respiratory support escalation (switch from HFNC to CPAP, NIV or intubation) or patient death because of worsening ARF. Finally, we analysed the factors associated with HFNC failure and in-hospital overall mortality.

In particular, we defined *failure* as the occurrence of one of the following: Device intolerance, Escalation of the ventilatory support in the ward (i.e. escalation from HFNC to CPAP or NIV), ICU admission, Need of intubation.

### Statistical analysis

Descriptive statistics were produced for demographic, clinical, and laboratory characteristics of patients. Mean and SD (or, in case of skewed distribution, median and interquartile range [IQR]) are reported for continuous variables, and number and percentages are reported for categorical variables.

The effect of HFNC on gas exchange, comfort, dyspnea, and respiratory rate was assessed through a mixed linear model for repeated measurements with clinical steps as predictors and subjects as random effects. In addition, post-hoc analysis through Tukey’s test was performed to detect variation on gas exchange, respiratory rate, dyspnea, and comfort before HFNC and after 2 and 24 h from its beginning.

Comparisons between patients’ cohorts (Death vs. Alive, Success vs. Failure) were performed with logistic regression. Odds ratios (OR) and associated 95% likelihood ratio-based confidence intervals were calculated. Multivariate analysis was not performed due to the low number of events.

All tests were two-sided, and p < 0.05 was chosen to indicate statistical significance. JMP version 15 software for Mac (SAS Institute, Cary, NC, USA) was used for statistical analysis.

Graphical representations were made with GraphPad Prism version 9.2.0 for Mac (GraphPad Software, San Diego, CA, USA).

## Results

**Fig. 2:**
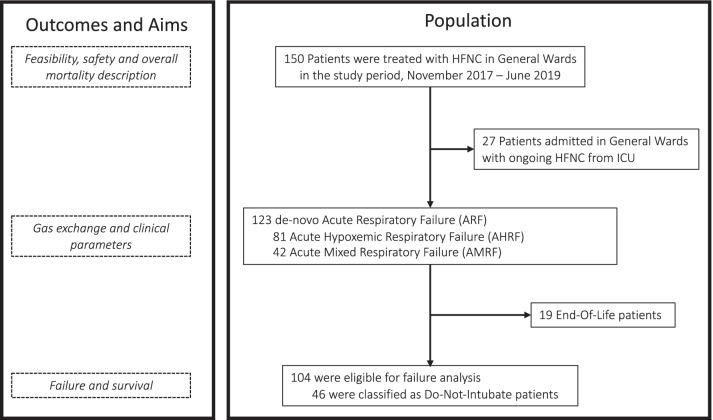
Patient population flowchart and related outcomes. *HFNC* high flow nasal cannula; *ICU* Intensive Care Unit

The study cohort and patient subgroups are presented in Fig. 2. From November 2017 to December 2019, 150 patients (81 male, 54.0%) were treated with HFNC in nine Hospital Wards and consequently included in the present study. The median age was 74 [60–80] years with a baseline SOFA score of 4 [2–4] and APACHE II score of 11 [8–13]. 123 patients (82.0%) had de novo ARF, while the remaining 27 (18.0%) were patients discharged from ICU with ongoing HFNC therapy. Overall, 85 patients (56.7%) were classified as candidate for full-code resuscitation, 46 (30.7%) as DNI, and 19 (12.6%) as EOL. The median time between ARF diagnosis and the start of HFNC was 4 [2–7] days; before HFNC, the most frequently used device was Venturi Mask (62 patients, 41.3%). The main cause of ARF was community-acquired pneumonia (71 patients, 48.0%), followed by hospital-acquired pneumonia (27 patients, 18.0%). In the latter case, the median time between hospital admission and pneumonia onset was 3 [2–10] days. Bilateral pulmonary infiltrates at chest X-ray were present in 65 patients (43.3%). Overall length of hospital stay was 22 [14–32] days. Patients’ characteristics are presented in Table 1 and Table S1 in Additional file 3 according to classification (i.e., Full Code resuscitation, DNI and EOL).

**Table 1:** Demographics and clinical characteristics of patients before HFNC start (n = 150)

Age—year	74 [60–80]
Male sex—no. (%)	81 (54%)
SOFA Score	4 [2–4]
APACHE II Score	11 [8–13]
Charlson’s Comorbidity Index	5 [3–6]
Comorbidities—no. (%)*	
COPD	52 (35%)
Cystic fibrosis	9 (6%)
Others respiratory	22 (15%)
Hypertension	47 (31%)
Malignancies	38 (25%)
Haematological	24 (15%)
Respiratory	7 (5%)
Others	7 (5%)
Cardiac	25 (17%)
Congestive heart failure	20 (13%)
Diabetes mellitus	17 (11%)
Neurologic	15 (10%)
Renal	10 (7%)
Hepatic	6 (4%)
Immunocompromised—no. (%)^§^	48 (32%)
Reason for hospital admission—no. (%)	
Respiratory	99 (66%)
Surgery	10 (7%)
Extrapulmonary sepsis	10 (7%)
Cardiac	6 (4%)
Mixed cardiac—respiratory	2 (1%)
Others	23 (15%)
Cause of acute respiratory failure—no. (%)	
Community-acquired pneumonia	71 (48%)
Hospital-acquired pneumonia	27 (18%)
COPD	15 (10%)
Others respiratory	17 (11%)
Cardiac failure	12 (8%)
Mixed cardiac—respiratory	8 (5%)
Bilateral infiltrates on chest radiograph—no. (%)	65 (43%)

*SOFA* Sequential Organ Failure Assessment; *APACHE II* Acute Physiologic Assessment and Chronic Health Evaluation; *COPD* chronic obstructive pulmonary disease

*Overlap may exist between comorbidities

^§^Immunocompromised: use of long-term (> 3 months) or high-dose (> 0.5 mg/kg/day) steroids, use of other immunosuppressant drugs, solid organ transplantation, solid cancer requiring chemotherapy in the last 5 years, hematologic malignancy regardless of time since diagnosis and received treatments, or primary immune deficiency

In the entire cohort of 150 patients, HFNC was applied for a total of 1399 days, and the median duration of treatment was 7 [3–11] days. We observed only one technical failure of the device and one dysfunction due to lack of water in the device humidification chamber. Both situations were promptly resolved without any impact on patients. No other adverse events or complications related to HFNC therapy were observed.

**Fig. 3:**
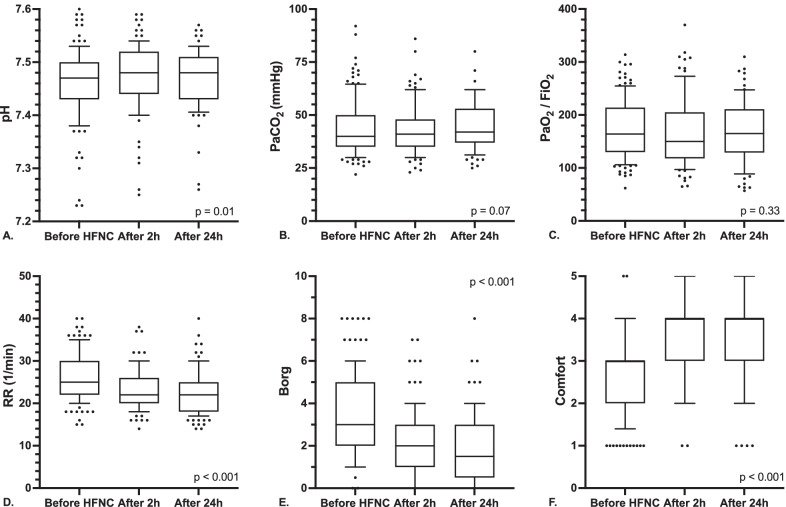
Gas exchange, respiratory rate, dyspnea and comfort before and during the first 24 h of HFNC oxygen therapy (n = 123 patients with de-novo ARF). **A** pH. **B** Arterial partial pressure of CO_2_ (PaCO_2_). **C** Arterial partial pressure of oxygen to inspiratory oxygen fraction ratio (PaO_2_/FiO_2_). **D** Respiratory rate (RR). **E** Borg dyspnea scale. **F** Comfort scale

The effects of HFNC on gas exchange, respiratory rate, dyspnea, and comfort was assessed in 123 patients with de novo ARF (i.e., after exclusion of the 27 patients discharged from the ICU with ongoing HFNC therapy) and are shown in Table 2 and Fig. 3. Median HFNC initial settings were: FiO_2_ 50%, flow 60 L/min and temperature 34 °C. Compared to baseline values, no significant changes of PaO_2_/FiO_2_ ratio and of PaCO_2_ were observed at 2 and 24 h after the start of HFNC. The median pH value increased from 7.47 to 7.48 (*p* = 0.01). Lactate levels remained stable at 1.5–1.4 mmol/L (p = 0.81), while Base Excess increased from 4.9 to 7.7 mmol/L (*p* = 0.21). Respiratory rate dropped from a median of 25 breaths/min before HFNC to 22 breaths/min both at 2 and 24 h (p  < 0.001). At 24 h, HFNC therapy led to a significant decrease in dyspnea Borg Scale (from 3 to 1 point, *p* < 0.001) and to an improvement of Comfort Scale (from 3 to 4 points, *p* < 0.001).

**Table 2:** Variations in breathing pattern, gas exchange, dyspnea and comfort before and during the first 24 h of HFNC oxygen therapy (n = 123)

	Before HFNC	After 2 h	After 24 h	p value
HFNC settings				
FiO_2_ (%)	40 [35–50]	50 [40–60]*	50 [36–60]*	< 0.001
Temperature (°C)	[–]	34 [31–37]	34 [31–37]	[–]
HFNC flow (L/min)	[–]	60 [50–60]	60 [50–60]	[–]
Arterial blood gases				
pH	7.47 [7.43–7.5]	7.48 [7.44–7.52]*	7.48 [7.43–7.51]	0.01
PaO_2_/FiO_2_ (mmHg)	164 [130–214]	150 [118–205]	165 [129–211]	0.33
PaO_2_ (mmHg)	69 [57–81]	74 [62–90]	69 [62–84]	0.06
PaCO_2_ (mmHg)	40 [35–50]	41 [35–48]	42 [37–53]	0.07
Lactate (mmol/L)	1.5 [1–2.1]	1.4 [1–2.1]	1.4 [1–2.1]	0.81
HCO_3_^−^ (mEq/L)	29 [25.9–33.2]	30.1 [27.05–35.7]	30.1 [26.6–34.7]*	0.09
BE (mmol/L)	4.9 [1.8–9.1]	5.9 [2.6–12.4]	7.7 [2.6–11.6]	0.21
Clinical data				
SpO_2_ (%)	95 [92–97]	96 [93–98]*	96 [93–98]	0.01
RR (breaths/min)	25 [22–30]	22 [20–26]*	22 [18–25]*	< 0.001
ROX Index	8.64 [6.6–11.99]	9.13 [6.74–11.25]	9.05 [6.72–12.47]	0.50
Borg Scale	3 [2–5]	2 [1–3]*	1 [0.5–3]*	< 0.001
Comfort Scale	3 [2, 3]	4 [3, 4]*	4 [3, 4]*	< 0.001

*HFNC* high flow nasal cannula; *FiO*_*2*_ inspired fraction of O_2_; *PaO*_*2*_ arterial partial pressure of O_2_; *PaCO*_*2*_ arterial partial pressure of CO_2_; *BE* base excess; *SpO*_*2*_ peripheral saturation of Hb; *RR* respiratory rate; *ROX Index* ratio of SpO_2_/FiO_2_ to respiratory rate

*p < 0.05 vs before HFNC

Among the 123 patients with de-novo ARF, 81 had AHRF and 42 had AMRF (Additional file 3: Tables S2, S3 and Additional file 1: Figures S1, Additional file 2: Figure S2). In both groups, we observed a statistically significant reduction of RR and dyspnea and an improvement of comfort at 2 and 24 h after HFNC. Notably, in AMRF patients, after 24 h of HFNC treatment the median PaCO_2_ remained stable and arterial pH slightly increased.

The rate of failure of HFNC therapy was assessed in 104 patients (i.e., after excluding the 19 EOL patients from the cohort of 123 patients with de novo ARF). Overall, HFNC failed in 20 patients (19.2%): 3 (2.9%) did not tolerate the device, 14 (13.4%) needed escalation of the ventilatory support to NIV or CPAP in the ward and 3 (2.9%) were transferred to ICU. Among the patients admitted to ICU, one continued HFNC and was discharged alive 8 days later, while the other 2 were intubated (respectively after 37 h and 44 h from the start of HFNC) and they both died. A comparison between patients who failed or succeeded HFNC is presented in Table 3. In the univariate analysis, patients who failed HFNC had a higher Charlson’s Comorbidity Index (odds ratio [OR] 1.29; 95% CI 1.07–1.55; p = 0.004), higher APACHE II Score (odds ratio [OR] 1.59; 95% CI 1.09–4.17; p = 0.003), and cardiac failure as cause of ARF (odds ratio [OR] 5.26; 95% CI 1.36–20.46; p = 0.02). In 12 patients ARF was due to cardiac failure, and they were treated with HFNC due to NIV/CPAP intolerance. 10 patients were included in the cohort of 104 patients with de-novo ARF and 5 of them failed.

**Table 3:** Characteristics and comparison of patients grouped by failure (n = 104)

	Success (n = 84)	Failure (n = 20)^#^	p value	OR (95% CI)
Age—year	76 [61–81]	75 [68–88]	0.15	1.02 (0.99–1.06)
Male sex—no. (%)	45 (54%)	7 (35%)	0.13	0.47 (0.17–1.28)
SOFA Score	3 [2–4]	4 [2–5]	0.21	1.18 (0.91–1.53)
APACHE Score	10 [7–14]	11 [10–15]	0.003	1.59 (1.09–4.17)
Charlson’s Comorbidity Index	4 [3–6]	6 [5–8]	0.004	1.29 (1.07–1.55)
Comorbidities—no. (%)*				
COPD	29 (35%)	9 (45%)	0.39	1.55 (0.58–4.17)
Others respiratory	22 (26%)	3 (15%)	0.27	0.50 (0.13–1.86)
Malignancies	15 (18%)	6 (30%)	0.24	1.97 (0.65–5.97)
Congestive heart failure	10 (12%)	3 (15%)	0.71	1.31 (0.32–5.26)
Cardiac	14 (17%)	5 (25%)	0.40	1.67 (0.52–5.33)
Hypertension	32 (38%)	4 (20%)	0.11	0.41 (0.12–1.32)
Hepatic	4 (5%)	1 (5%)	0.96	1.05 (0.11–9.96)
Renal	7 (8%)	1 (5%)	0.60	0.58 (0.07–4.99)
Diabetes mellitus	7 (8%)	2 (10%)	0.81	1.22 (0.23–6.38)
Neurologic	8 (10%)	2 (10%)	0.95	1.05 (0.21–5.40)
Immunocompromised—no. (%)^§^	20 (24%)	6 (30%)	0.57	1.37 (0.46–4.04)
Reason for hospital admission—no. (%)				
Respiratory	63 (75%)	12 (60%)	0.19	0.5 (0.18–1.39)
Surgery	5 (6%)	1 (5%)	0.87	0.83 (0.09–7.54)
Extrapulmonary sepsis	3 (4%)	2 (10%)	0.27	3 (0.47–19.28)
Cardiac	3 (4%)	2 (10%)	0.27	3 (0.47–19.28)
Mixed cardiac—respiratory	1 (1%)	0 (0%)	0.51	[–]
Others	9 (10%)	3 (15%)	0.60	1.47 (0.36–6.02)
Cause of acute respiratory failure—no. (%)				
Community-acquired pneumonia	43 (51%)	9 (45%)	0.62	0.78 (0.29–2.08)
Hospital-acquired pneumonia	13 (16%)	3 (15%)	0.96	0.96 (0.25–3.76)
COPD	11 (13%)	2 (10%)	0.70	0.73 (0.15–3.62)
Others respiratory	6 (7%)	1 (5%)	0.72	0.68 (0.08–6.03)
Cardiac failure	5 (6%)	5 (25%)	0.02	5.26 (1.36–20.46)
Mixed cardiac—respiratory	6 (7%)	0 (0%)	0.10	[–]
Bilateral infiltrates in chest radiograph—no. (%)	29 (35%)	11 (55%)	0.09	2.32 (0.86–6.23)
Death—no (%)	6 (7%)	10 (50%)^$^	< 0.001	13 (3.89–43.48)

*FiO*_*2*_ inspired fraction of O_2_; *PaO*_*2*_ arterial partial pressure of O_2_; *ROX Index* ratio of SpO_2_/FiO_2_ to respiratory rate

*Overlap may exist between comorbidities

^§^Immunocompromised: use of long-term (> 3 months) or high-dose (> 0.5 mg/kg/day) steroids, use of other immunosuppressant drugs, solid organ transplantation, solid cancer requiring chemotherapy in the last 5 years, hematologic malignancy regardless of time since diagnosis and received treatments, or primary immune deficiency

^#^Of the 20 patients who failed, 3 did not tolerate the device, 14 need escalation of the ventilatory support in the ward and only 3 were transferred to ICU. 2 patients out of the 3 admitted to ICU died for ARF worsening. Hence, the rate of failure is 20/104 (19.2%)

^$^Of the 10 patients who died, 8 had a do-not-intubate order and only 2 had been assigned a “full code resuscitation”

The overall mortality rate in HFNC failure group was 50% and the odds ratio for in-hospital death was 13 times higher in the failure group (95% CI 3.89–43.48, p < 0.001).

Worsening of PaO_2_/FiO_2_, dyspnea Borg scale, comfort and ROX Index after 2 and 24 h of HFNC were associated with increased risk of failure (Additional file 3: Table S4).

Forty-two out of 150 patients died and the overall in-hospital mortality was 28%. Of these 42 patients, 19 were EOL, 19 were DNI (8 of whom died after HFNC failure for worsening ARF), 2 died for surgical complications (unrelated to ARF) and 2 died for ARF in ICU. Among patients who failed HFNC, overall mortality rate was 50% (10 patients of 20 died). Analyzing the 16 patients who died in the cohort of 104 patients (i.e., after excluding the 19 EOL patients from the of 123 patients with de novo ARF), higher SOFA Score (odds ratio [OR] 1.55; 95% CI 1.16–2.08; p = 0.002), higher APACHE II Score (odds ratio [OR] 3.21; 95% CI 1.15–10.09; p < 0.001), and surgical reason for hospital admission (odds ratio [OR] 6.54; 95% CI 1.19–35.91; p = 0.04) were associated with a higher risk of death (Table 4).

**Table 4:** Characteristics and comparison of Patients grouped by outcome (n = 104)

	Alive (n = 88)	Death (n = 16)	p value	OR (95% CI)
Age—year	76 [61–82]	76 [61–86]	0.48	1.01 (0.98–1.05)
Male sex—no. (%)	45 (51%)	7 (44%)	0.59	0.74 (0.25–2.17)
SOFA Score	3 [2–4]	5 [3–7]	0.002	1.55 (1.16–2.08)
APACHE II Score	11 [8–13]	12 [10–15]	< 0.001	3.21 (1.15–10.09)
Charlson’s Comorbidity Index	5 [3–6]	5 [3–6]	0.66	0.54 (0.03–9.36)
Comorbidities—no. (%)*				
COPD	31 (35%)	7 (44%)	0.52	1.43 (0.49–4.21)
Others respiratory	22 (25%	3 (19%)	0.58	0.69 (0.18–2.66)
Malignancies	16 (18%)	5 (31%)	0.25	2.05 (0.62–6.71)
Congestive heart failure	13 (15%)	0 (0%)	0.03	[–]
Cardiac	15 (17%)	4 (25%)	0.46	1.62 (0.46–5.72)
Hypertension	30 (34%)	6 (38%)	0.79	1.16 (0.38–3.50)
Hepatic	3 (3%)	2 (13%)	0.17	4.05 (0.62–26.43)
Renal	5 (6%)	3 (19%)	0.11	3.83 (0.82–17.98)
Diabetes mellitus	7 (8%)	2 (13%)	0.57	1.65 (0.31–8.79)
Neurologic	9 (10%)	1 (6%)	0.60	0.59 (0.07–4.97)
Immunocompromised—no. (%)^§^	21 (24%)	5 (31%)	0.53	1.45 (0.45–4.65)
Reason for hospital admission—no. (%)				
Respiratory	67 (76%)	8 (50%)	0.04	0.31 (0.10–0.94)
Surgery	3 (3%)	3 (19%)	0.04	6.54 (1.19–35.91)
Sepsis	3 (3%)	2 (12%)	0.17	4.04 (0.62–26.43)
Cardiac	5 (7%)	0 (0%)	0.19	[–]
Mixed cardiac—respiratory	1 (1%)	0 (0%)	0.56	[–]
Others	9 (10%)	3 (19%)	0.35	2.02 (0.48–8.48)
Cause of acute respiratory failure—no. (%)				
Community-acquired pneumonia	44 (50%)	8 (50%)	1	1 (0.34–2.90)
Hospital-acquired pneumonia	14 (16%)	2 (12%)	0.72	0.76 (0.15–3.70)
COPD	10 (11%)	3 (19%)	0.43	1.80 (0.44–7.43)
Others respiratory	6 (7%)	1 (7%)	0.93	0.91 (0.10–8.12)
Cardiac failure	8 (9%)	2 (12%)	0.68	1.43 (0.27–7.44)
Mixed cardiac	6 (7%)	0 (0%)	0.15	[–]
Bilateral infiltrates in chest radiograph—no. (%)	33 (38%)	7 (43%)	0.63	1.30 (0.44–3.81)
Failure—no (%)^#^	10 (11%)	10 (62%)^$^	< 0.001	13 (3.89–43.48)

*Overlap may exist between comorbidities

^§^Immunocompromised: use of long-term (> 3 months) or high-dose (> 0.5 mg/kg/day) steroids, use of other immunosuppressant drugs, solid organ transplantation, solid cancer requiring chemotherapy in the last 5 years, hematologic malignancy regardless of time since diagnosis and received treatments, or primary immune deficiency

^#^Of the 20 patients who failed, 3 did not tolerate the device, 14 need escalation of the ventilatory support in the ward and only 3 were transferred to ICU. 2 patients out of the 3 admitted to ICU died for ARF worsening. Hence, the rate of failure is 20/104 (19.2%)

^$^Of the 10 patients who died, 8 had a do-not-intubate order and only 2 had been assigned a “full code resuscitation”

## Discussion

The main findings of this study can be summarized as follows. Treatment of mild-to-moderate ARF patients with HFNC outside the ICU was feasible and seamed safe after an initial training and under the daily supervision of an intensivist. HFNC support did not significantly affect gas exchanges but was associated with an improvement in comfort, dyspnea, and respiratory rate. Of note, these effects were detectable already after 2 h of HFNC and confirmed at the daily monitoring in all patients in whom HFNC therapy was successful. Finally, HFNC failed in less than 20% of our cohort.

The application of non-invasive respiratory supports—including HFNC—in settings that lack intensive monitoring (such as hospital general wards) has been largely debated. On the one hand, it is well known that HFNC has potential benefits compared to standard oxygen [8], however delayed recognition of failure can expose the patients to a risk of late intubation, which in turn results in worse outcome [9]. On the other hand, ICU admission of every patient with mild to moderate ARF is impossible in most Countries, due to the limited availability of ICU beds. To allow an early application of HFNC in patients with ARF admitted to general wards while avoiding delayed recognition of failure, we developed a strategy based on: (1) an educational project for doctors and nurses of the general wards and (2) daily monitoring performed by an intensivist of our Outreach Team of all patients treated with HFNC outside the ICU.

Before the COVID-19 pandemic, data on the use of HFNC outside the ICU were extremely scarce. Lenglet et al. firstly described the use of heated and humified HFNC in the emergency department on 17 patients with ARF, in whom HFNC was associated with by better comfort, reduction in RR and improved oxygenation compared to conventional oxygen therapy [3]. Similar results have been reported by Zemach et al., who observed that HFNC determined greater improvement in dyspnea in patients with a history of respiratory disease or higher pre-connection dyspnea [4]. Recently, Jackson et al. reported on the use of HFNC outside the ICU in a cohort of 346 patients with AHRF after specific training in the wards, showing for the first time that appropriate patient selection and staff education are essential for a safe and effective application of HFNC in “less protected environments” [6]. For this reason, in our study, the educational meetings were targeted both to medical and nursing staff and the application of specific protocols for monitoring and recognition of failure was mandated.

During the recent COVID-19 pandemic, clinicians have been forced to treat with noninvasive respiratory supports, including HFNC, a large number of patients with AHRF. Franco and Guy analyzed the feasibility and clinical impact of non-invasive respiratory supports (HFNC, Helment CPAP and NIV) outside the ICU during the pandemic [10, 11]. Recently, Issa et al. reported on the safety and feasibility of HFNC oxygenation therapy as primary treatment and after ICU stabilization in COVID-19 patients. The authors emphasized how the use of HFNC can be effective in hypoxic patients, reducing the workload for already overburdened ICUs [12].

Initially, HFNC was recommended only in hypoxemic patients without hypercapnia, but recent experiences have demonstrated that HFNC may be effective also in chronically hypercapnic patients [13, 14]. However, a recent multicenter, non-inferiority randomized trial conducted in COPD patients showed that HFNC may be inferior to NIV as initial strategy of ventilatory support [15].

In our study, the application of HFNC in the subgroup of patients with AMRF did not result in worsening of hypercapnia and was associated with a significant improvement of respiratory rate and dyspnea. However, we have to acknowledge that these patients had mild and compensated hypercapnia, as demonstrated by pH values within the normal range.

Finally, in our patient population, the application of HFNC in general wards had a relatively low rate of failure, with only 3% of the patients needing ICU admission. This very low failure rate can be attributed to multiple factors: (1) patient selection; (2) specific training to doctors and nurses in the hospital wards; (3) daily supervision and monitoring by an intensivist. HFNC failure was associated with worsening of PaO_2_/FiO_2_, dyspnea Borg scale, comfort and ROX Index after 2 and 24 h of HFNC and, notably, none of the patients who failed had an improvement of RR, comfort or dyspnea, suggesting that HFNC success is related to its effect on both patient comfort and gas exchange. In a previous study on patients with ARF, we showed that HFNC settings (flow and temperature) have a significant impact on patient comfort, with higher flows being associated with improved comfort only in the more severely hypoxemic patients [16].

This study has several limitations. First, due to the study design and the relatively low sample size, we were unable to adjust our analysis for potential confounders, and we provide only raw data, comparisons, and univariate associations. Thus, our analysis should be considered explorative in nature. Second, our study cohort was quite heterogeneous in terms of diagnosis and characteristics. However, this allowed us to test the efficacy and safety of HFNC applied in general wards in a ‘real world’ scenario. Third, our study did not include a control group, so we could not perform a comparison with other forms of respiratory support, nor validate the effectiveness of the whole strategy (i.e. training and supervision). Fourth, the heterogeneity of the patient population (in particular regarding the etiology of ARF and the reversibility of the clinical condition) may have an important impact on the interpretation of our results, since the aim of the use of HFNC should be different depending on patients’ conditions. Finally, all participating wards belong to the same Hospital, which limits the external validity of our findings, that should be confirmed in larger, multicenter studies.

## Conclusions

In patients with mild to moderate hypoxemic and hypoxemic-hypercapnic ARF admitted to general wards, the use of HFNC after an initial training and daily supervision by intensivists was feasible, and seemed to be safe. HFNC was effective in improving comfort, dyspnea and respiratory rate, without significant effects on gas exchanges even in patients with mild hypercapnia. Moreover, it was associated with a low risk of failure and ICU admission. Further studies are needed to evaluate this strategy in larger patient populations from different centers.

## Supplementary Information

**Additional file 1: Figure S1.** Gas exchange, respiratory rate, dyspnea and comfort before and during the first 24 h of HFNC Oxygen Therapy in pure hypoxemic ARF patients (AHRF) (n = 81).

**Additional file 2: Figure S2.** Gas exchange, respiratory rate, dyspnea and comfort before and during the first 24 h of HFNC Oxygen Therapy in mixed hypoxemic-hypercapnic ARF patients (AMRF) (n = 42).

**Additional file 3: Table S1.** Demographics and clinical characteristics of patients according to classification. **Table S2.** Variations in breathing pattern, gas exchange, dyspnea and comfort before and during the first 24 h of HFNC Oxygen Therapy in pure hypoxemic ARF patients (AHRF) (n = 81). **Table S3.** Variations in breathing pattern, gas exchange, dyspnea and comfort before and during the first 24 h of HFNC Oxygen Therapy in mixed hypoxemic-hypercapnic ARF patients (AMRF) (n = 42). **Table S4.** Predictors of failure during the first 24 h of HFNC Oxygen Therapy in ARF patients (n = 104).

## Data Availability

Data were recorded on an local electronic worksheet, personal data has been completely anonymized. The datasets used and/or analysed during the current study are available from the corresponding author on reasonable request.

## Abbreviations

AHRF: Acute hypoxemic respiratory failure
AMRF: Acute mixed respiratory failure
APACHE II: Acute Physiologic Assessment and Chronic Health Evaluation II Score
ARF: Acute respiratory failure
COVID-19: Coronavirus-19 disease
CPAP: Continuous Positive Airways Pressure
DNI: Do Not Intubate
EOL: End Of Life
FiO_2_: Inspiratory oxygen fraction
GCS: Glasgow Coma Scale
HFNC: High flow nasal cannula
ICU: Intensive Care Unit
NIV: Non Invasive Ventilation
PaCO_2_: Arterial partial pressure of carbon dioxide
PaO_2_: Arterial partial pressure of oxygen
PaO_2_/FiO_2_: Arterial partial pressure of oxygen to inspiratory oxygen fraction ratio
RR: Respiratory rate
SOFA: Sequential Organ Failure Assessment Score
SpO_2_: Pulse oximetry
