# Supplementary figures and images for: Use of high flow nasal cannula in patients with acute respiratory failure in general wards under intensivists supervision: a single center observational study
Source: Respir Res. 2022 Jun 26;23:171. doi: 10.1186/s12931-022-02090-x (PMC9233759; doi:10.1186/s12931-022-02090-x)

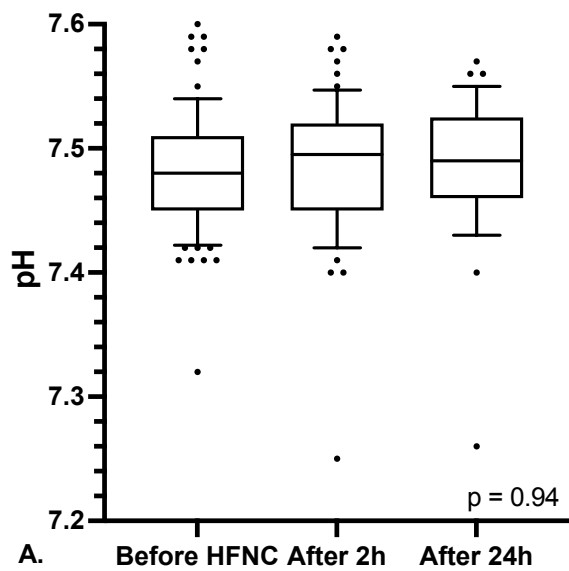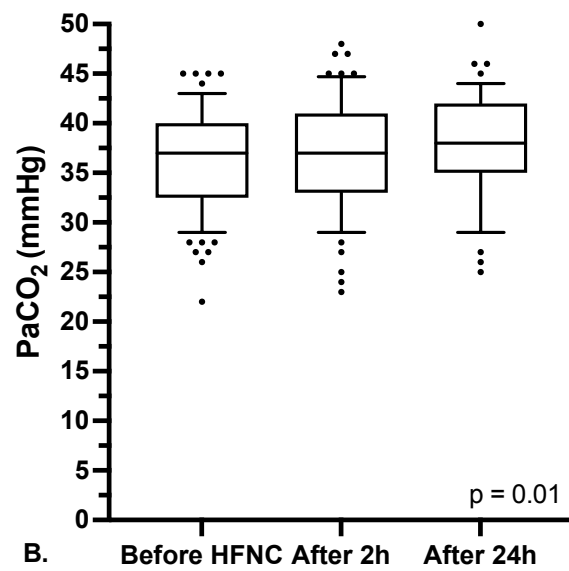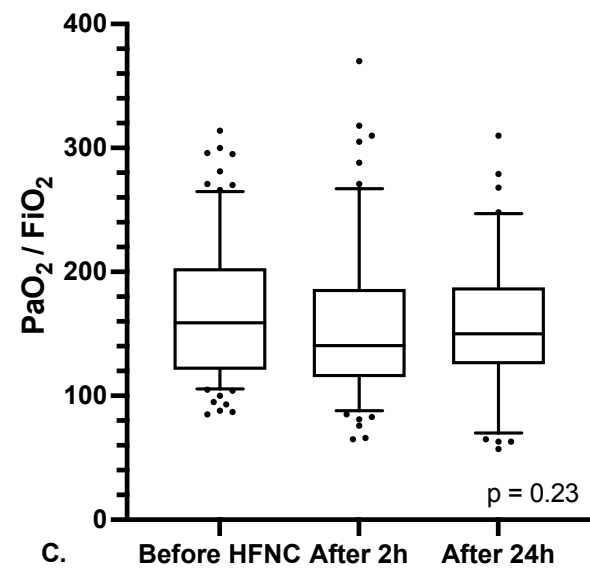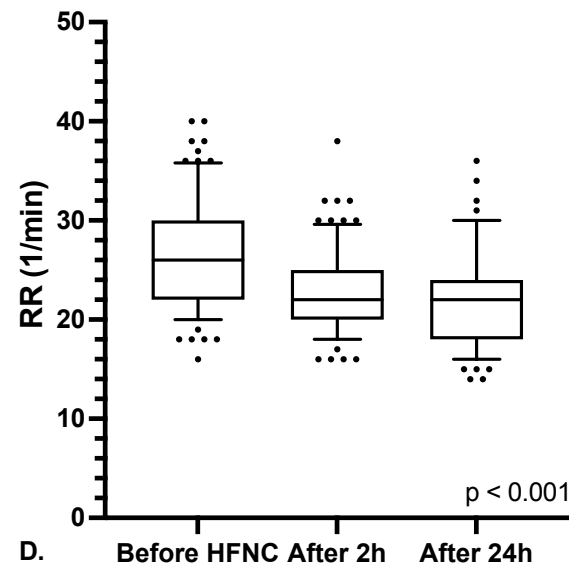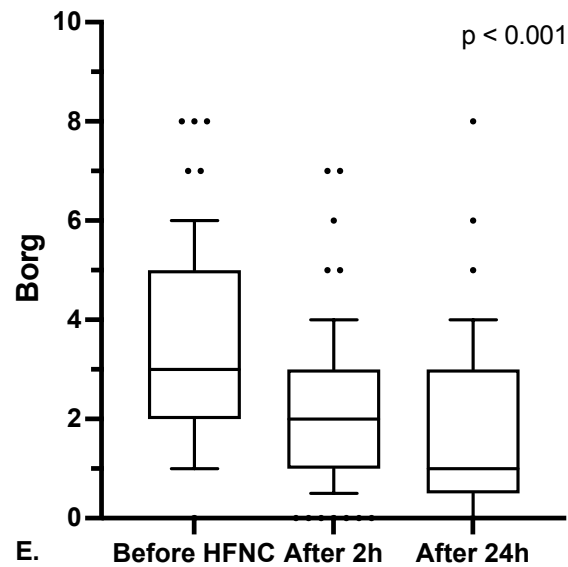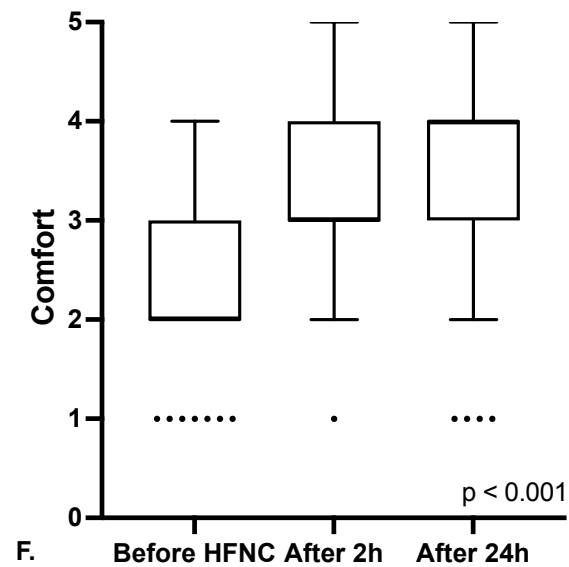

Supplement: Supplementary file 1 — Additional file 1: Figure S1. Gas exchange, respiratory rate, dyspnea and comfort before and during the first 24 h of HFNC Oxygen Therapy in pure hypoxemic ARF patients (AHRF) (n = 81). [file 12931_2022_2090_MOESM1_ESM.pdf]

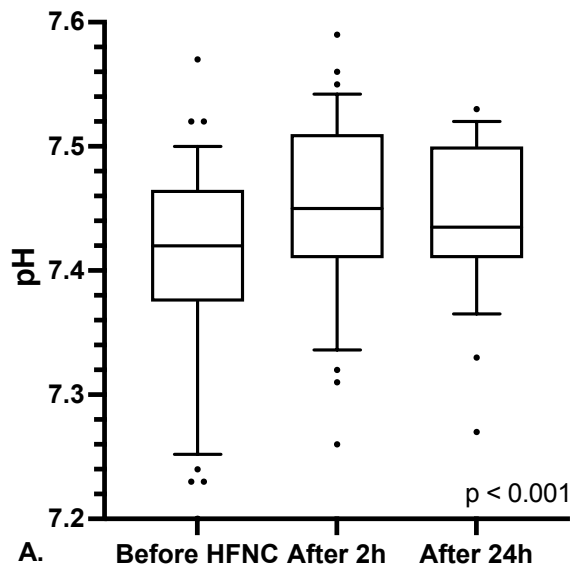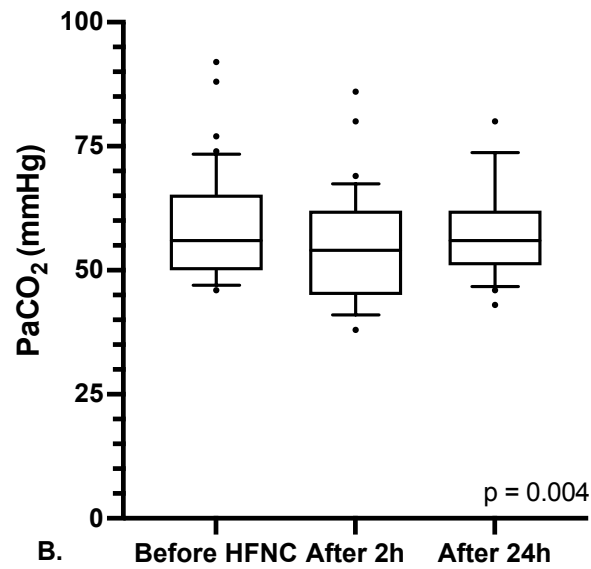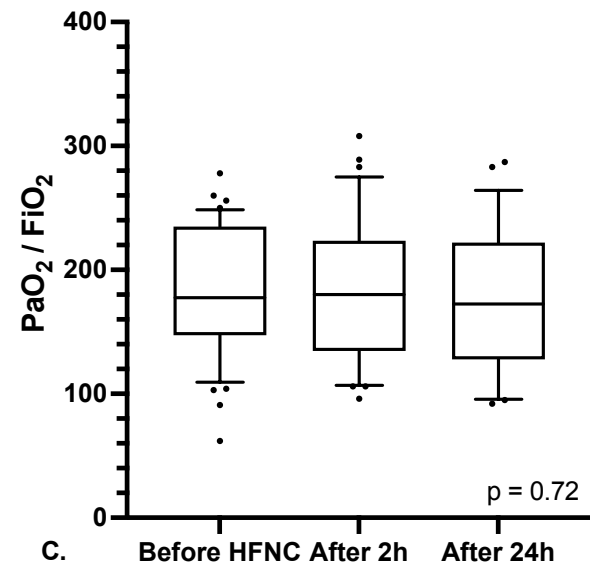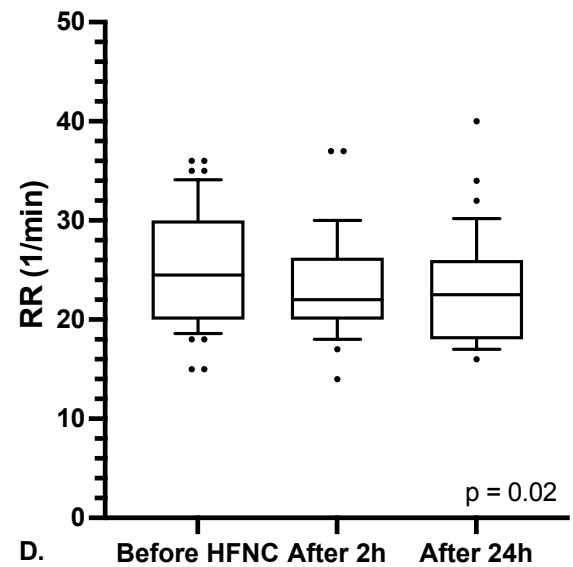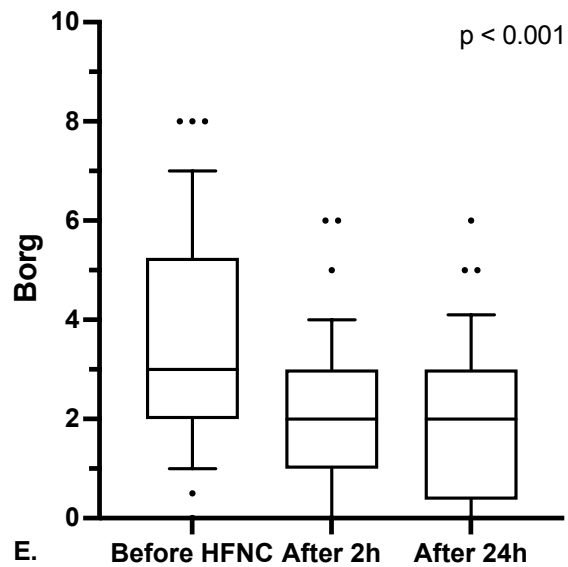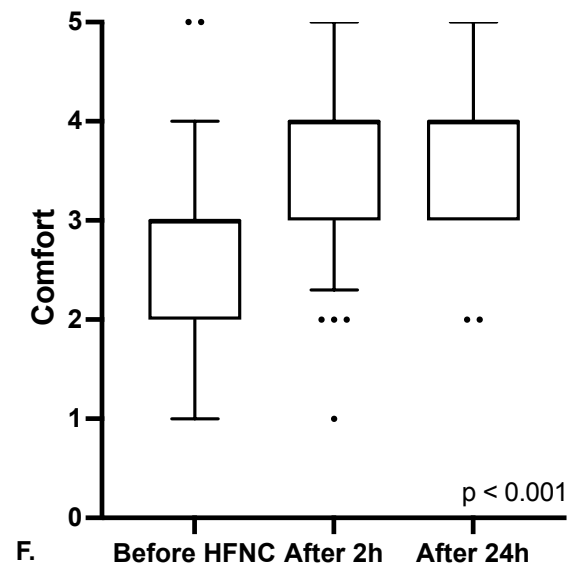

Supplement: Supplementary file 2 — Additional file 2: Figure S2. Gas exchange, respiratory rate, dyspnea and comfort before and during the first 24 h of HFNC Oxygen Therapy in mixed hypoxemic-hypercapnic ARF patients (AMRF) (n = 42). [file 12931_2022_2090_MOESM2_ESM.pdf]
