# Supplementary material for: Use of high flow nasal cannula in patients with acute respiratory failure in general wards under intensivists supervision: a single center observational study
Source: Respir Res. 2022 Jun 26;23:171. doi: 10.1186/s12931-022-02090-x (PMC9233759; doi:10.1186/s12931-022-02090-x)
Supplement: Supplementary file 3 — Additional file 3: Table S1. Demographics and clinical characteristics of patients according to classification. Table S2. Variations in breathing pattern, gas exchange, dyspnea and comfort before and during the first 24 h of HFNC Oxygen Therapy in pure hypoxemic ARF patients (AHRF) (n = 81). Table S3. Variations in breathing pattern, gas exchange, dyspnea and comfort before and during the first 24 h of HFNC Oxygen Therapy in mixed hypoxemic-hypercapnic ARF patients (AMRF) (n = 42). Table S4. Predictors of failure during the first 24 h of HFNC Oxygen Therapy in ARF patients (n = 104). [file 12931_2022_2090_MOESM3_ESM.docx]

**Use of High Flow Nasal Cannula in patients with Acute Respiratory Failure in general wards under intensivists supervision: a single center observational study**

**Authors:**

Sebastiano Maria Colombo^1,2^, Vittorio Scaravilli^1,3^, Andrea Cortegiani^4,5^, Nadia Corcione^6,7^, Amedeo Guzzardella^2^, Luca Baldini^8,9^, Elisa Cassinotti^10^, Ciro Canetta^8^, Stefano Carugo^8,11^, Cinzia Hu^8^, Anna Ludovica Fracanzani^2,12^, Ludovico Furlan^8,11^, Maria Chiara Paleari^1,2^, Alessandro Galazzi^13^, Paola Tagliabue^1^, Flora Peyvandi^2,8^, Francesco Blasi^2,14^, and Giacomo Grasselli^1,2^

**Authors’ affiliations:**

1. Fondazione IRCCS Ca' Granda Ospedale Maggiore Policlinico, Department of Anaesthesia and Intensive Care Medicine, Milan, Lombardia, IT
2. University of Milan, Department of Pathophysiology and Transplantation, Milan, Lombardia, IT
3. University of Milan, Department of Department of Biomedical, Surgical and Dental Sciences Milan, Lombardia, IT
4. University of Palermo, Department of Surgical, Oncological and Oral Science (Di.Chir.On.S.), Palermo, Sicilia, IT
5. Policlinico Paolo Giaccone, Department of Anesthesia, Intensive Care and Emergency, Palermo, Sicilia, IT
6. Fondazione IRCCS Ca' Granda Ospedale Maggiore Policlinico, Respiratory Unit and Cystic Fibrosis Adult Center, Milan, Lombardia, IT
7. Azienda Ospedaliera Antonio Caldarelli, Interventional Pulmunology, Naples, Campania, IT
8. Fondazione IRCCS Ca' Granda Ospedale Maggiore Policlinico, Department of Internal Medicine, Milan, Lombardia, IT
9. University of Milan, Department of Oncology and Hemato-oncology, Milan, Lombardia, IT
10. Fondazione IRCCS Ca' Granda Ospedale Maggiore Policlinico, Department of Surgery, Milan, Lombardia, IT
11. University of Milan, Department of Clinical Sciences and Community Health, Milan, Lombardia, IT
12. Fondazione IRCCS Ca' Granda Ospedale Maggiore Policlinico, Unit of Internal Medicine and Metabolic Disease, Milan, Lombardia, IT
13. Fondazione IRCCS Ca' Granda Ospedale Maggiore Policlinico, Healthcare Profession Department, Milan, Lombardia, IT
14. Fondazione IRCCS Ca' Granda Ospedale Maggiore Policlinico, Internal Medicine Department and Respiratory Medicine Unit, Milan, Lombardia, IT

# Methods

*Training courses*

Specific training courses have been conducted before initiating HFNC support in general wards. Theoretical lesson lasting about 2 hours were addressed to doctors and nurses of the general wards.

Main topics covered were:

- Theoretical approach to acute respiratory failure
- Respiratory support modality
- HFNC functioning
- HFNC physiological effects
- HFNC initiation and maintenance
- Early identification of failure
- Evaluation of the shared protocol with intensivists

Practical approaches were then conducted in subgroups, explaining how to assemble the circuit and how to deal with the main technical failures. Finally, the lessons were periodically repeated to consolidate the concepts learned.

*Surveillance*

As per standard management and guidelines within the hospital, the patients to nurse ratio, patients to doctor ratio and staff rounds varied between wards according to the intensity of care provided. In particular:

- Patients to nurse ratio ranged between 1:6 to 1:10 daily, while up to 1:20 overnight
- Specific ward attending physicians were available daily, while an interdivisional on call-physician overnight
- Nurses rounds were usually divided in 3 shifts/day: morning, afternoon and nightshifts
- The intensivist of the Outreach Team evaluated daily the patients included in the study (i.e, once a day or more according to the patient's condition) by conducting a complete physical examination and blood gas analysis. Further indications regarding patients’ management and HFNC support have been daily discussed with the ward staff. 24/24hrs not dedicated on-call physician was available in the hospital for any request.

# Results

| **Table S1. Demographics and clinical characteristics of patients according to classification** | | | |
| --- | --- | --- | --- |
|  | Full Code  (85 pts, 56.7%) | DNI  (46 pts, 30.7%) | EOL  (19 pts, 12.6%) |
| Age – yr | 72 [55 – 78] | 71 [62 – 75] | 79 [72 – 88] |
| Male sex – no. (%) | 50 (58.8%) | 22 (47.8%) | 9 (47.4%) |
| SOFA Score | 3 [2 – 4] | 4 [2 – 5] | 4 [2 – 6] |
| APACHE II Score | 10 [6 – 13] | 11 [10 – 14] | 12 [9 – 13] |
| Charlson’s Comorbidity Index | 4 [3 – 6] | 5 [3 – 7] | 7 [4 – 9] |
| Comorbidities – no. (%) * | |  |  |
| COPD | 34 (40.0%) | 13 (28.3%) | 5 (26.3%) |
| Cystic Fibrosis | 7 (8.2%) | 2 (4.3%) | 0 (0.0%) |
| Others respiratory | 11 (12.9%) | 7 (15.2%) | 4 (21.1%) |
| Hypertension | 19 (22.4%) | 21 (45.7%) | 7 (36.8%) |
| Malignancies | 14 (16.5%) | 12 (26.1%) | 12 (63.2%) |
| Haematological | 12 (14.1%) | 8 (17.4%) | 4 (21.1%) |
| Respiratory | 1 (1.2%) | 2 (4.3%) | 4 (21.1%) |
| Others | 1 (1.2%) | 2 (4.3%) | 4 (21.1%) |
| Cardiac | 10 (11.8%) | 8 (17.4%) | 7 (36.8%) |
| Congestive heart failure | 11 (12.9%) | 7 (15.2%) | 2 (10.5%) |
| Diabetes mellitus | 7 (8.2%) | 8 (17.4%) | 2 (10.5%) |
| Neurologic | 7 (8.2%) | 6 (13.0%) | 2 (10.5%) |
| Renal | 5 (5.9%) | 4 (8.7%) | 1 (5.3%) |
| Hepatic | 2 (2.4%) | 4 (8.7%) | 0 (0.0%) |
| Immunocompromised – no. (%) ^§^ | 18 (21.2%) | 14 (30.4%) | 16 (84.2%) |
| Reason for hospital admission – no. (%) | |  |  |
| Respiratory | 60 (70.6%) | 29 (63.0%) | 10 (52.6%) |
| Surgery | 5 (5.9%) | 4 (8.7%) | 1 (5.3%) |
| Extrapulmonary Sepsis | 6 (7.1%) | 3 (6.5%) | 1 (5.3%) |
| Cardiac | 2 (2.4%) | 3 (6.5%) | 1 (5.3%) |
| Mixed cardiac – respiratory | 1 (1.2%) | 0 (0.0%) | 1 (5.3%) |
| Others | 11 (12.9%) | 6 (13.0%) | 6 (31.6%) |
| Cause of Acute Respiratory Failure – no. (%) | |  |  |
| Community-acquired Pneumonia | 43 (50.6%) | 22 (47.8%) | 6 (31.6%) |
| Hospital-acquired Pneumonia | 10 (11.8%) | 7 (15.2%) | 10 (52.6%) |
| COPD | 10 (11.8%) | 5 (10.9%) | 0 (0.0%) |
| Others respiratory | 13 (15.3%) | 2 (4.3%) | 2 (10.5%) |
| Cardiac Failure | 5 (5.9%) | 7 (15.2%) | 0 (0.0%) |
| Mixed cardiac – respiratory | 5 (5.9%) | 2 (4.3%) | 1 (5.3%) |
| Bilateral infiltrates on chest radiograph – no. (%) | 34 (40.0%) | 20 (43.5%) | 11 (57.9%) |
| Patients’ classification: Full code (Full Code Resuscitation), DNI (Do Not Intubate), EOL (End Of Life)  * Overlap may exist between comorbidities. % are expressed as ratio within the group  § Immunocompromised: use of long-term (>3 months) or high-dose (>0.5 mg/kg/d) steroids, use of other immunosuppressant drugs, solid organ transplantation, solid cancer requiring chemotherapy in the last 5 years, hematologic malignancy regardless of time since diagnosis and received treatments, or primary immune deficiency.  *SOFA*: Sequential Organ Failure Assessment; *COPD*: Chronic Obstructive Pulmonary Disease. | | | |

| **Table S2. Variations in breathing pattern, gas exchange, dyspnea and comfort before and during the first 24 hours of HFNC Oxygen Therapy in pure hypoxemic ARF patients (AHRF) (n = 81).** | | | | |
| --- | --- | --- | --- | --- |
|  | Before HFNC | After 2h | After 24h | p-value |
| HFNC Settings | | | | |
| FiO_2_ (%) | 50 [35-56] | 50 [43.5-60] * | 52 [40-60] * | < 0.001 |
| HFNC Temperature (°C) | [-] | 34 [31-37] | 34 [31-37] | [-] |
| HFNC Flow (L/min) | [-] | 60 [50-60] | 60 [50-60] | [-] |
| Arterial Blood Gases | | | | |
| pH | 7.48 [7.45-7.51] | 7.50 [7.45-7.52] | 7.49 [7.46-7.53] | 0.94 |
| PaO_2_/FiO_2_ (mmHg) | 159 [121-203] | 141 [115-186] | 150 [126-188] | 0.23 |
| PaO_2_ (mmHg) | 71 [58-85] | 77 [69-92] * | 75 [66-93] | 0.04 |
| PaCO_2_ (mmHg) | 37 [33-40] | 37 [33-41] | 38 [35-42] * | 0.01 |
| Lactate (mmol/L) | 1.7 [1.1-2.5] | 1.4 [1.1-2.6] | 1.5 [1-2.2] | 0.73 |
| HCO_3_^-^ (mEq/L) | 27.2 [25-29.1] | 28 [25-30.5] | 28.9 [25.6-32] * | 0.05 |
| Base Excess (mmol/L) | 3.3 [1.2-5.7] | 4.4 [0.6-6.7] | 5.2 [1.8-9.4] | 0.81 |
| Physical Data | | | | |
| SpO_2_ (%) | 95 [93-98] | 97 [94-98] | 97 [94-98] | 0.18 |
| RR (breaths/min) | 26 [22-30] | 22 [20-25] * | 22 [18-24] * | < 0.001 |
| ROX Index | 8.2 [6.33-10.89] | 8.71 [6.17-10.1] | 8.78 [6.28-12.11] | 0.25 |
| Borg Scale | 3 [2-5] | 2 [1-3] * | 1 [0.5-3] * | < 0.001 |
| Comfort Scale | 2 [2-3] | 3 [3-4] * | 4 [3-4] * | < 0.001 |
| *) p < 0.05 vs Before HFNC.  *HFNC*: High Flow Nasal Cannula; *FiO_2_*: inspired fraction of O_2_; *PaO_2_*: arterial partial pressure of O_2_; *PaCO_2_*: arterial partial pressure of CO_2_; *Hb*: haemoglobin; *SpO_2_*: peripheral saturation of Hb; *RR*: respiratory rate; *ROX Index*: ratio of SpO_2_/FiO_2_ to respiratory rate. | | | | |

| **Table S3. Variations in breathing pattern, gas exchange, dyspnea and comfort before and during the first 24 hours of HFNC Oxygen Therapy in mixed hypoxemic-hypercapnic ARF patients (AMRF) (n = 42).** | | | | | | |  |
| --- | --- | --- | --- | --- | --- | --- | --- |
|  | | Before HFNC | After 2h | After 24h | p-value | | |
| HFNC Settings |  |  |  |  |  |  |  |
| FiO_2_ (%) | | 38 [28-50] | 38 [31-50] | 36 [34-50] | 0.97 | | |
| HFNC Temperature (°C) | | [-] | 34 [31-37] | 34 [31-37] | [-] | | |
| HFNC Flow (L/min) | | [-] | 52 [50-60] | 55 [50-60] | [-] | | |
| Arterial Blood Gases |  |  |  |  |  |  |  |
| pH | | 7.42 [7.38-7.47] | 7.45 [7.41-7.51] * | 7.44 [7.41-7.5] * | < 0.001 | | |
| PaO_2_/FiO_2_ (mmHg) | | 178 [147-235] | 180 [135-224] | 173 [128-222] | 0.72 | | |
| PaO_2_ (mmHg) | | 64 [54-78] | 68 [59-81] | 65 [59-74] | 0.75 | | |
| PaCO_2_ (mmHg) | | 56 [50-65] | 54 [45-62] * | 56 [51-62] | 0.004 | | |
| Lactate (mmol/L) | | 1.3 [0.8-1.8] | 1.3 [0.8-1.8] | 1.2 [0.7-1.8] | 0.61 | | |
| HCO_3_^-^ (mEq/L) | | 35.7 [32-39.4] | 36 [32.3-41.4] | 37 [34.2-41.2] | 0.12 | | |
| Base Excess (mmol/L) | | 9.7 [7.2-15.8] | 12.5 [8.3-17.1] | 12.4 [11.3-18.7] | 0.04 | | |
| Physical Data |  |  |  |  |  |  |  |
| SpO_2_ (%) | | 94 [91-96] | 95 [92-97] * | 95 [93-96] * | 0.01 | | |
| RR (breaths/min) | | 25 [20-30] | 22 [20-26] * | 23 [18-26] * | 0.02 | | |
| ROX Index | | 9.18 [7.96-13.66] | 9.75 [8.89-14.48] | 10.44 [7.75-14.41] | 0.62 | | |
| Borg Scale | | 3 [2-5] | 2 [1-3] * | 2 [0.5-3] * | < 0.001 | | |
| Comfort Scale | | 3 [2-3] | 4 [3-4] * | 4 [3-4] * | < 0.001 | | |
| *) p < 0.05 vs Before HFNC.  *HFNC*: High Flow Nasal Cannula; *FiO_2_*: inspired fraction of O_2_; *PaO_2_*: arterial partial pressure of O_2_; *PaCO_2_*: arterial partial pressure of CO_2_; *Hb*: haemoglobin; *SpO_2_*: peripheral saturation of Hb; *RR*: respiratory rate; *ROX Index*: ratio of SpO_2_/FiO_2_ to respiratory rate. | | | | | |  |  |

| **Table S4. Predictors of failure during the first 24 hours of HFNC Oxygen Therapy in ARF patients (n = 104).** | | | | | |
| --- | --- | --- | --- | --- | --- |
|  | Success  (n=84) | | Failure  (n=20) | p-value | OR (95% CI) |
| Δ_1_ PaO_2_/FiO_2_ | | 3 ± 62 | - 40 ± 68 | 0.01 | 0.98 (0.97 – 0.99) |
| Δ_2_ PaO_2_/FiO_2_ | | 3 ± 61 | - 56 ± 63 | 0.003 | 0.98 (0.97 – 0.99) |
| Δ_1_ Borg | | -2 ± 2 | 0 ± 1 | < 0.001 | 2.12 (1.35 - 3.55) |
| Δ_2_ Borg | | - 2 ± 2 | 0 ± 1 | < 0.001 | 2.23 (1.43 - 3.49) |
| Δ_1_ Comfort | | 1 ± 1 | 0 ± 1 | 0.01 | 0.50 (0.28 - 0.90) |
| Δ_2_ Comfort | | 1 ± 1 | 0 ± 2 | 0.008 | 0.62 (0.43 - 0.90) |
| Δ_1_ ROX Index | | 0.37 ± 3.91 | - 2.13 ± 4.01 | 0.02 | 0.86 (0.88 - 0.97) |
| Δ_2_ ROX Index | | 0.43 ± 4.47 | - 2.3 ± 5.75 | 0.03 | 0.90 (0.82 - 0.99) |
| Rox Index > 5.7 at 2 h | | 79 (94%) | 15 (75%) | 0.019 | 0.19 (0.05 – 0.74) |
| Rox Index < 5.7 at 24 h | | 5 (6%) | 5 (25%) | 0.019 | 5.27 (1.36 – 20.46) |
| Rox Index > 4.88 at 2 h | | 83 (99%) | 16 (80%) | 0.002 | 0.05 (0.01 – 0.46) |
| Rox Index < 4.88 at 24 h | | 1 (1%) | 4 (20%) | 0.002 | 20.75 (2.17 – 197.99) |
| *Δ_1_*: difference between measurement at 2 h after HFNC and before HFNC; *Δ_2_*: difference between measurement at 24 h after HFNC and before HFNC. | | | | | |

**FIGURE LEGENDS**

**Figure S1**

**Gas exchange, respiratory rate, dyspnea and comfort before and during the first 24 hours of HFNC Oxygen Therapy in pure hypoxemic ARF patients (AHRF) (n = 81).**

*Panel A: pH; Panel B: Arterial partial pressure of CO_2_ (PaCO_2_); Panel C: arterial partial pressure of oxygen to inspiratory oxygen fraction ratio (PaO₂/FiO₂); Panel D: Respiratory rate (RR); Panel E: Borg dyspnea scale; Panel F: Comfort scale*

**Figure S2**

**Gas exchange, respiratory rate, dyspnea and comfort before and during the first 24 hours of HFNC Oxygen Therapy in mixed hypoxemic-hypercapnic ARF patients (AMRF) (n = 42).**

*Panel A: pH; Panel B: Arterial partial pressure of CO_2_ (PaCO_2_); Panel C: arterial partial pressure of oxygen to inspiratory oxygen fraction ratio (PaO₂/FiO₂); Panel D: Respiratory rate (RR); Panel E: Borg dyspnea scale; Panel F: Comfort scale*
